# Twenty-Five Years' Institutional Progress

**Published:** 1911-10-07

**Authors:** 


### The Hospital

The Workers' Newspaper of Administrative Medicine and Institutional Life, Administration, National Insurance and Health.

No. 1317, Vol. LI. SATURDAY, OCTOBER 7TH, 1911. PRICE ONE PENNY.

### TWENTY-FIVE YEARS' INSTITUTIONAL PROGRESS.

### THE PAST AND THE FUTURE.

On September 30, 1911, The Hospital completed the first quarter of a century of its existence. In its first number we chose as our motto Lowell's words: "Be noble, and the nobleness that lies in other men, sleeping, but never dead, will rise in majesty to meet thine own." The work it was founded to further is still as ennobling as, but infinitely greater in volume than, it was twenty-five years ago. The workers have increased at least tenfold in the twenty-five years that have passed, and their keenness has grown with the ever-extending knowledge and efficiency which mark the doing of most work for the sick in the present day.

The aim of The Hospital has always been to extend the area of public interest and knowledge in the work of those who labour amongst sickness and suffering and the dependents of all classes. When The Hospital commenced its work twenty-five years ago we were compelled to state plainly that the public conscience was then undoubtedly asleep in regard to such matters. It will startle many to be reminded that we were writing at a period when hospital hygiene was far to seek and the mortality after serious operations amounted to at least one death among every three persons so treated. Trained nurses, as we know them to-day, were yet in the making. At that time the maximum standard for the highest qualified nurses was one year's training in a recognised nursing school. We had to urge upon all who wished to become nurses that every nurse must secure this training, for a certificate from such a school was the only guarantee of knowledge and efficiency that she could obtain. Many of our oldest and most famous hospitals were doing their work in buildings which had long since become inefficient for the purpose and urgently called for reconstruction. There was an absence of intercommunication and of accurate data for the enforcement of an average standard of administration, and urgent need for the opening of many new departments fully equipped with the best appliances for successful treatment. The Metropolitan hospitals especially were starved for want of money, and had collectively each year a deficiency of some £100,000, which increased to £140,000 per annum within the succeeding decade. Those who devoted their lives to nursing the sick were practically without any means of securing a pension and sickness allowance when occasion arose—and the work which devolved upon them was still to a large extent menial, and so in conflict with the higher duties of devoted attendance upon the sick. The sleeping and other accommodation provided for the resident staff and the nurses was frequently lamentable in its inefficiency, and the hours of duty and the absence of systematic provision for relaxation and leisure were lamentable, too. We might add many other defects which called for reform.

Only twenty-five years have passed, but they have produced the modern hospital. What, then, has the modern up-to-date voluntary hospital of to-day given to the people in substitution for the evils we have enumerated? We can speak from accurate knowledge after personal inspection, and the facts are incontrovertible. Hospital mortality has fallen from 33 per cent. to something like 3 per cent. in major surgical cases, whilst the number of operations has increased nearly tenfold. The buildings of every great hospital, almost without exception, have been reconstructed, reorganised, and extended until to-day the best of them are the most perfectly organised, magnificently equipped, and efficiently staffed that the world has ever possessed. The multiplication of new departments, the introduction of electrical and mechanical aids to treatment, and the enforcement of cleanliness, are the marvel and pride of every educated mind charged with sufficient knowledge to appreciate their perfection. The King's Hospital Fund, established in 1897, will ever be one of the most glorious monuments of the recognition on the part of a great King of the privilege to the healthy of personal service for the sick. This Fund has freed the Metropolitan hospitals from the annual burden of £140,000 deficiency, and has so stimulated interest in the voluntary hospitals and their work that the ordinary income of the London hospitals has increased by 44 per cent., of the Irish hospitals by 53 per cent., of the provincial hospitals by 40 per cent., and of the Scotch hospitals by 36 per cent. Economically the King's Fund has restrained ill-considered expenditure upon bricks and mortar and produced a saving in the expenditure of certain voluntary hospitals in London which approaches £45,000 per annum. Our voluntary hospitals and the public generally have available an army of fully-trained certificated nurses, who have the safeguards and comfort of the Royal National Pension Fund, which enables them to obtain sick allowances and pensions and has become the greatest thrift agency amongst women workers in the world. Special provision has also been made for pensions for mental, fever and other attendants on the sick. The nurses are now provided by every hospital which is entitled to be regarded as a modern up-to-date institution with blocks of buildings where each nurse has a separate bedroom, and where the accommodation for their recreation and education is as perfect and complete as knowledge and money can make it. The average efficiency of the accommodation for all members of the resident staff in our best hospitals has continuously improved, and a visit to a modern hospital to-day is one of the most inspiriting and helpful experiences which an educated man or woman can enjoy. Indeed, all institutions for the sick and dependent have progressed steadily in the period under review, for we have included under the word "hospital" mental, Poor Law, fever, and convalescent establishments.

The Hospital can, therefore, look back over a quarter of a century with encouragement, and look forward with justifiable hope. The marvellous developments which we have briefly indicated have resulted in the recruiting of an army of institutional workers trained, competent, enthusiastic, and proud of their profession. It has now come to be recognised that every member of the staff of a modern hospital to attain the standard of efficiency required for success must have been thoroughly trained for the multifarious duties, involving in many cases intimate technical knowledge, nowadays so essential to successful work. Service in a hospital is a sine quâ non to the attainment of the highest success in all branches of the medical profession. Equal service and training are essential for the superintendent or house governor, the secretary, the matron, and every other official down to the humblest wardmaid, if they are to be equal to the duties attaching to their office, and capable of possessing the spirit of devotion which such work everywhere demands. It follows that efficient institutional workers seek to maintain their knowledge, to keep in touch with every development, with the trend of current opinion and the never-ending demands of scientific development.

In such circumstances, after twenty-five years' experience and active co-operation in practically every field of progress which has contributed to the perfecting of our institutions, we realise the help it must prove to the profession and the public to be provided with accurate and practical information concerning their progress and work. It is naturally the wish of the institutional workers to possess a newspaper devoted to their interests, where they can find just the information they require, and, by co-operation with its Editor, through correspondence and inquiry, make it complete in every department. The work of development which has produced the modern hospital and the expert institutional official of all grades is now fairly complete. We have, therefore, determined to devote our chief energies for the future to the provision of a newspaper for the institutional workers of the world which, with their assistance, we hope to make so complete in a few months' time as to make it contribute to the maintenance of high efficiency in all branches of the hospital field, whilst it becomes for the institutional worker not only a helpful companion but a tower of strength.

